# Sex discordance in asthma and wheeze prevalence in two longitudinal cohorts

**DOI:** 10.1371/journal.pone.0176293

**Published:** 2017-04-25

**Authors:** Ryan Arathimos, Raquel Granell, John Henderson, Caroline L. Relton, Kate Tilling

**Affiliations:** 1 School of Social and Community Medicine, University of Bristol, Bristol, United Kingdom; 2 Medical Research Council / University of Bristol Integrative Epidemiology Unit, University of Bristol, Bristol, United Kingdom; National Taiwan University College of Public Health, TAIWAN

## Abstract

Sex discordance in asthma prevalence has been previously reported, with higher prevalence in males before puberty, and in females after puberty; the adolescent “switch”. However, cross-sectional studies have suggested a narrowing of this discordance in recent decades. We used a combination of cross-sectional and longitudinal modelling to examine sex differences in asthma, wheeze and longitudinal wheezing phenotypes in two UK birth cohorts, the Avon Longitudinal Study of Parents and Children (ALSPAC; born 1991–92 with data from age 0–18 years) and the Millennium Cohort Study (MCS; born 2000–02 with data from age 3–10 years). We derived measures of asthma and wheeze from questionnaires completed by mothers and cohort children. Previously-derived ALSPAC wheezing phenotype models were applied to MCS. Males had a higher prevalence of asthma at 10.7 years in ALSPAC (OR 1.45 95%CI: 1.26, 1.66 n = 7778 for current asthma) and MCS (OR 1.42 95%CI: 1.29, 1.56 n = 6726 for asthma ever) compared to females, decreasing in ALSPAC after puberty (OR 0.94 95%CI: 0.79, 1.11 n = 5023 for current asthma at 16.5 years). In longitudinal models using restricted cubic splines, males had a clear excess for asthma in the last 12 months and wheeze in the last 12 months up until 16.5 years of age in ALSPAC. Males had an increased risk of all derived longitudinal wheezing phenotypes in MCS when compared to never wheeze and no evidence of being at lower risk of late wheeze when compared to early wheeze. By comparing data in two large, contemporary cohorts we have shown the persistence of sex discordance in childhood asthma, with no evidence that the sex discordance is narrowing in recent cohorts.

## Introduction

Higher incidence and prevalence of asthma have been previously reported in males in childhood and in females in adolescence and adulthood [1–3]. The age of this gender switch has not been precisely determined, with conflicting research on whether pubertal stages or puberty are associated with the switch in prevalence [1, 4, 5]. These differences appear to stem from biological differences between sexes as well as sociocultural and environmental differences. The biological differences can be further broken down into genetic, pulmonary (including pulmonary developmental) and immunological differences [6].

An analysis of the Isle of Wight (IOW) birth cohort identified males as being at higher risk of asthma and wheeze up until 10 years of age, with a switch in prevalence occurring by 18 years. The study acknowledged that wheeze and asthma are related traits, with wheeze often being a precursor to asthma development and thus focused on the transition dynamics between the two conditions. The positive transition from wheeze to asthma appeared higher among boys between the ages of 4 and 10 years compared to girls, whereas it was negative among boys and positive among girls between 10 and 18 years of age [7].

The most extensive international asthma survey performed thus far was the International Study of Asthma and Allergies in Childhood (ISAAC), which included 463,801 children aged 13–14 years old across 56 countries and 257,800 children aged 6–7 years in 38 countries. The study concluded that the prevalence of asthma was greater in males in the 6–7 age group, whereas females had higher prevalence in the 13–14 age group, with considerable variation between countries [3].

Some recent studies have failed to find a clear difference in asthma prevalence between sexes or have observed a narrowing of the sex-discordance in recent years [8–10]. A study from 2007 reports a narrowing of the sex ratio in school-age children aged 9–11 years over calendar time. Data was collected over a 15 year period, comprising four cross-sectional questionnaires completed in 1989, 1994, 1999 and 2004 by the parents of children at selected public schools in Scotland. Although the study demonstrated a sex discordance in the 1989 and the 1994 surveys, it did not detect a difference between sexes in 1999 and 2004 [8], indicating that although sex difference in asthma prevalence existed in previous generations, it seemed to no longer be the case in individuals born in the last two decades. Studies that have not shown a clear sex discordance in asthma may be suffering from inadequate power or poor study design.

In the present study, we look at the sex discordance, and time-trends in sex discordance, of asthma by comparing data from two large, contemporary UK birth cohorts recruited a decade apart [11]. We investigated whether the increased male prevalence is indeed attenuating in recent years as some previous studies have indicated[8, 9].We analysed the sex discordance in prevalence of asthma and wheeze in The Avon Longitudinal Study of Parents and Children (ALSPAC, recruited 1991–1992), a birth cohort where study children have reached puberty and the Millennium Cohort Study (MCS, recruited 2000–2002) a more recent cohort where the study children have not yet transitioned through puberty but we may still expect to find a higher prevalence of males with asthma in early childhood.

## Methods

### ALSPAC

The Avon Longitudinal Study of Parents and Children (ALSPAC) recruited 14,541 pregnant women resident in Avon, UK with expected dates of delivery 1st April 91 to 31st December 92. The initial number of pregnancies was 14,541, for which the mother enrolled in the ALSPAC study and had either returned at least one questionnaire or attended a “Children in Focus” clinic by 19/07/99.

When the oldest children were approximately 7 years of age, an attempt was made to bolster the initial sample with participants who had failed to join the study originally. The number of new pregnancies not in the initial sample was 706, resulting in an additional 713 children being enrolled.

The total sample size for analyses using any data collected after the age of seven is therefore 15,247 pregnancies, resulting in 15,458 fetuses. Of this total sample of 15,458 fetuses, 14,775 were live births and 14,701 were alive at 1 year of age.

The phases of enrolment are described in detail in the cohort profile paper [12]. The study website contains details of all the data that is available through a searchable data dictionary http://www.bris.ac.uk/alspac/researchers/data-access/data-dictionary/.

Informed written consent was obtained from the parents after receiving a complete description of the study at the time of enrolment into the ALSPAC project, with the option for them or their children to withdraw at any time. Ethical approval for the study was obtained from the ALSPAC Law and Ethics Committee and the Local Research Ethics Committees.

#### ALSPAC data

Reports of children’s wheezing were obtained from questionnaires sent to the mothers at approximately annual intervals from 0.5 (6 months) to 16.5 years. We used answers to the questions “Has your study child had wheezing or whistling on the chest in the past 12 months?” at 0.5, 1.5, 2.5, 3.5, 4.8, 5.8, 6.8, 7.6, 8.6, 10.7, 13.8 and 16.5 years and “Has your child had asthma in the past 12 months?” at 6.8, 7.6, 8.6, 10.7, 13.1 and 13.8 years.

We combined the data from the mother completed questionnaires with further data from child completed questionnaires, beginning at 16.5 years, where study children were asked the same questions about asthma and wheeze symptoms in the past 12 months. At 18.6 years study children were only asked about wheezing in the past 12 months. A full breakdown of questionnaires time-points can be seen in Table A in S1 Appendix.

For measures of doctor’s diagnosis of asthma we used answers obtained from study questionnaires completed by the mothers of the study children at 7.6, 10.7 and 13.8 years of age and by study children at 16.5 years of age. The questions asked were “Has a doctor ever actually said that your study child has asthma?” and “Have you or your parents ever been told by a doctor that you have asthma?” respectively for the mother-completed and child-completed questionnaires.

From the core ALSPAC sample of 14,701 alive at 1 year of age, 7,544 were listed as male and 7,155 as female (with 2 participants unknown/not listed), of which 7,323 males and 6,836 females were from the initial ALSPAC sample.

The study website contains details of all the data that are available through a fully searchable data dictionary at < www.bris.ac.uk/alspac/researchers/data-access/data-dictionary/ >.

### Millennium Cohort Study (MCS)

The MCS is a nationally representative cohort study of 18,818 infants from 18,553 families born in the UK. A random two-stage sample of all infants born in the UK between 2000 and 2002, and who were alive and resident in the UK at 9 months was drawn from the Department of Social Security Child Benefit Registers. Children born in England and Wales were recruited between September 2000 and August 2001, and children born in Scotland and Northern Ireland were recruited between 2000 and 2002. Child Benefit Registers cover virtually all children, but exclude those whose residence status is either uncertain or temporary. Children who had died within the first 9–10 months of life were excluded (less than 1% of all births). The study used stratified sampling by electoral ward, with oversampling to ensure adequate representation of families living in poverty and those living in areas with high ethnic minority populations. Parents and guardians were interviewed by trained interviewers to capture sociodemographic and health information when their children were 9 months old, with follow-ups at approximately 3[13], 5[14], 7[15] and 10 years[11, 16]. Informed written consent was sought from parents for their participation and the participation of their child/children at each MCS survey. Where parents gave consent to the participation of their child/children in one or more elements of a survey the inclusion of the child/children requires their agreement and compliance. As with the other Centre for Longitudinal Studies (CLS) cohorts and irrespective of any consent or assent, individuals are able to refuse to participate in any element of a survey or withdraw from the study at any time by simply expressing the wish to do so.

#### MCS data

Parents and guardians were interviewed by trained interviewers to capture sociodemographic and health information when their children were 9 months old, with follow-ups at approximately 3, 5, 7 and 10 years. We used data from the follow-ups at 3, 5, 7 and 10 years, from a total of 8850 males and 8458 females, where the ISAAC questionnaire for asthma was included in the interview. The ISAAC questionnaire has been previously used and validated and includes questions on both asthma and wheeze symptoms [17].

### Statistical analysis

Logistic regressions were performed for the outcomes asthma and wheeze in the last 12 months from both the mother-completed and the child-completed questionnaires in ALSPAC. Child-completed data were used at 18.6 years where there was no mother-completed data available for wheeze and at 16.5 years when there was no mother-completed data for asthma. In the MCS, we used reports of asthma ever, wheeze ever and wheeze in the last 12 months. Based on guidance of the MCS datasets, issued by the Centre for Longitudinal Studies, we included the stratum design variable of the MCS in regression models and adjusted for clustering by ward.

We considered whether any variables should be included as confounders in our examination of the association between sex and wheeze/asthma. The definition of a confounder is a variable that causes both the dependent variable and the independent variable[18, 19]. Thus in our example, any potential confounder would have to be a cause of the child’s sex at birth (the exposure). Therefore, we consider sex (the exposure) to be independent of any external influences or confounding, since the only determinants of a child’s sex are the meiotic processes subject to Mendels law of independent assortment. Thus we have not included any potential confounding variables in this analysis.

**Fig 1:**
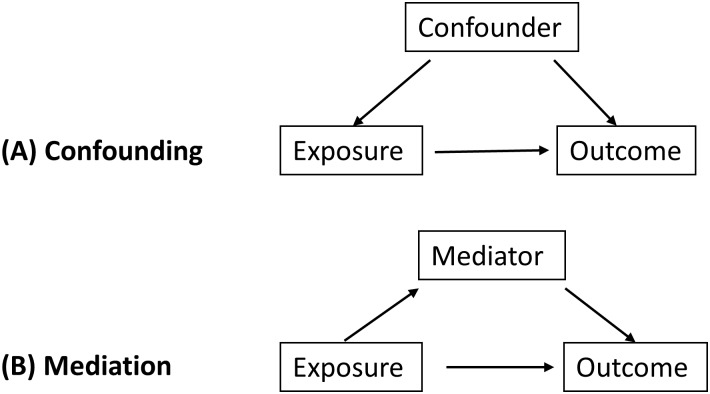
Concepts of (A) confounding and (B) mediation in epidemiological studies illustrated using a Directed acyclic graph (DAG). In the case of sex and asthma, where sex is the exposure and asthma status the outcome, any extraneous factors must go by the mediation pathway, as nothing can influence a child’s sex at conception.

Factors such as infant respiratory infection rates, parental healthcare-seeking behaviour, sport activities, onset of smoking/obesity and age at puberty (or other factors which often vary by sex) could not possibly be confounders, but could be on the causal pathway and may mediate how the sex of the child affects their asthma/wheeze status [18]. Adjusting for such variables could lead to collider bias, and therefore these variables have not been included in any of the models [20]. We have used a Directed acyclic graph (DAG) to assess and illustrate this concept (Fig 1).

#### Longitudinal model

Longitudinal logistic regression models were used in ALSPAC and the MCS to address the problem of different samples being taken at different time-points. The models were fitted using MLwiN v2.34[21, 22] and STATA 13.1/MP2[23]. We did not assume that the relationship between asthma/wheeze and age was linear, and so fit all models using restricted cubic splines, in order to allow for the most flexible shape and circumvent issues with model fit. In ALSPAC, for wheeze in the last 12 months we used 5 knots points at ages 0.5, 3.5, 6.8, 10.7, 16.7 years (based on Harrell’s recommended percentiles[24]). For asthma in the last 12 months we used 3 knots at ages 6.8, 10.8, 16.7 years as there were only 7 time-points of data available at which asthma status was assessed. We performed a sensitivity analysis where we used 5 knots instead of 3 knots and found no major differences between the asthma models (data available from authors). We used both the mother-completed and child-completed data for wheeze in the last 12 months (up to 18.6 years) and for asthma in the last 12 months (up to 16.5 years). We included the MCS stratum design variable as a covariate and took into account potential clustering by study ward. We used indicator variables to account for differences in the mother vs child completed data at overlapping time-points. In the MCS, we used restricted cubic splines with 3 knots at ages 3.1, 5.5, 11.3 years for both wheeze ever and asthma ever and at 3, 5.3, 11.1 years for wheeze in the last 12 months (both as specified by Harrell’s recommended percentiles[24]).

#### Wheezing phenotypes

Six wheezing phenotypes (never/infrequent, transient early, prolonged early, intermediate onset, late onset and persistent wheezing) were previously identified in ALSPAC using Latent class analysis (LCA) and based on longitudinal patterns of reported wheezing up to 7 years of age [25]. All phenotypes found in ALSPAC, with the exception of prolonged early wheezing were also previously identified in an independent Dutch cohort (PIAMA) [26]. We replicated this analysis in the MCS data, using the same methods. We also performed an additional sensitivity analysis where we stratified the LCA model by sex and generated wheezing phenotypes specific to each sex in the MCS, in order to determine if the same wheezing phenotypes generated from the full sample were the best fit for females and males separately. This analysis mirrors the sex stratified analysis performed in a recent ALSPAC study that identified wheezing phenotypes extended to approximately 16.5 years of age [27].

For the MCS analysis, we assumed 3 phenotypes in an initial model, and compared models with increasing numbers of phenotypes using the Bayesian Information Criterion (BIC), the model entropy and the Lo-Mendell-Rubin adjusted likelihood ratio tests (LRT) [28] in participants with complete responses to questionnaires about 12 month wheezing (N = 7,349). In this analysis we did not weight by stratum design variable and did not take potential clustering by ward into account, as there was no evidence from our other models of clustering impacting the model results. We estimated the conditional probabilities of wheezing in the optimal model at each of the 4 time points, in addition to the posterior probabilities of phenotype membership for each child. In our results we use and discuss the conditional probabilities for each identified phenotype. All analyses were done using Mplus 7.1 software [29] and STATA 13.1/MP2[23].

## Results

Characteristics of the data used in both the cross-sectional analysis and the longitudinal models for ALSPAC are shown in Table 1.

**Table 1:** Characteristics of wheeze and asthma outcomes in ALSPAC by sex, with follow-up rates at each time-point as a percentage of total cohort size for each sex (total ALSPAC = 7,544 males and 7,155 females).

Age (years)		Asthma in last 12 months*		Wheeze in last 12 months	
		N with data (% follow-up)	n with asthma (% of N)	N with data (% follow-up)	n with wheeze (% of N)
**Mother completed** **time-points (years)**					
**0.5**	Male	-	-	5884(78.0%)	1759(29.9%)
	Female	-	-	5510(77.0%)	1243(22.6%)
**1.5**	Male	-	-	5662(75.1%)	1744(30.8%)
	Female	-	-	5291(73.9%)	1272(24.0%)
**2.5**	Male	-	-	5157(68.4%)	1321(25.6%)
	Female	-	-	4812(67.3%)	936(19.5%)
**3.5**	Male	-	-	5166(68.5%)	1000(19.4%)
	Female	-	-	4817(67.3%)	757(15.7%)
**4.8**	Male	-	-	4862(64.4%)	1038(21.3%)
	Female	-	-	4528(63.3%)	739(16.3%)
**5.8**	Male	-	-	4429(58.7%)	767(17.3%)
	Female	-	-	4156(58.1%)	561(13.5%)
**6.8**	Male	4365(57.9%)	609(14.0%)	4315(57.2%)	646(15.0%)
	Female	4125(57.7%)	454(11.0%)	4071(56.9%)	481(11.8%)
**7.6**	Male	4215(55.9%)	568(13.5%)	4220(55.9%)	531(12.6%)
	Female	3984(55.7%)	387(9.7%)	3989(55.8%)	353(8.8%)
**8.6**	Male	4179(55.4%)	572(13.7%)	4172(55.3%)	629(15.1%)
	Female	4033(56.4%)	402(10.0%)	4024(56.2%)	427(10.6%)
**10.7**	Male	3914(51.9%)	545(13.9%)	3850(51.0%)	568(14.8%)
	Female	3864(54.0%)	389(10.1%)	3804(53.2%)	368(9.7%)
**11.7**	Male	-	-	3693(49.0%)	509(13.8%)
	Female	-	-	3710(51.9%)	363(9.8%)
**13.1**	Male	3492(46.3%)	473(13.5%)	3486(46.2%)	431(12.4%)
	Female	3526(49.3%)	360(10.2%)	3518(49.2%)	311(8.8%)
**13.8**	Male	3537(46.9%)	452(12.8%)	3476(46.1%)	427(12.3%)
	Female	3507(49.0%)	366(10.4%)	3464(48.4%)	338(9.8%)
**16.5**	Male	-	-	2736(36.3%)	286(10.5%)
	Female	-	-	2905(40.6%)	307(10.6%)
**Child completed** **time-points (years)**					
**16.5**	Male	2055(27.2%)	245(11.9%)	2047(27.1%)	353(17.2%)
	Female	2968(41.5%)	374(12.6%)	2960(41.4%)	668(22.6%)
**18.6**	Male	-	-	1189(15.8%)	185(15.6%)
	Female	-	-	2162(30.2%)	470(21.7%)

*Questions on asthma in the last 12 months were not included in ALSPAC questionnaires between the ages of 6 months and 5.8 years, at 11.5 years and at 18.6 years.

Characteristics of the variables in the MCS defining wheeze and asthma by sex are shown in Table 2.

**Table 2:** Characteristics of wheeze and asthma outcomes in the MCS by sex, with follow-up rates at each time-point as a percentage of total cohort size for each sex (total MCS = 8850 males and 8458 females).

Age (years)		Ever asthma†		Ever wheeze†		Wheeze in last 12 months*†	
		N with data (% follow-up)	n with asthma (% of N)	N with data (% follow-up)	n with wheeze (% of N)	N with data (% follow-up)	n with wheeze (% of N)
**3.1**	Male	7731(87.4)	1137 (14.8)	7866(88.9)	2689 (34.2)	7866(88.9)	1749 (22.3)
	Female	7453(88.2)	764 (10.3)	7563(89.5)	2062 (27.3)	7563(89.5)	1332 (17.7)
**5.2**	Male	7732(87.4)	1380 (17.9)	7753(87.7)	2647 (34.2)	7752(87.6)	1456 (18.8)
	Female	7380(87.3)	931 (12.7)	7410(87.7)	1896 (25.6)	7409(87.6)	1039 (14.1)
**7.2**	Male	6979(78.9)	1343 (19.3)	6993(79.1)	2153 (30.8)	6992(79.1)	1000 (14.4)
	Female	6767(80.1)	927 (13.7)	6781(80.2)	1534 (22.7)	6780(80.2)	676 (10)
**10.7**	Male	6646(75.1)	1366 (20.6)	6652(75.2)	1644 (24.8)	6652(75.2)	926 (14)
	Female	6519(77.1)	1011 (15.6)	6518(77.1)	1211 (18.6)	6517(77.1)	650 (10)

* Wheeze in the last 12 months is a subset question of ever wheeze as laid out in the cohort questionnaire. Negative controls were incorporated from the answers to the ever wheeze question.

^†^ Values are not adjusted for MCS sampling weights.

### Cross-sectional prevalence of asthma and wheeze

**Fig 2:**
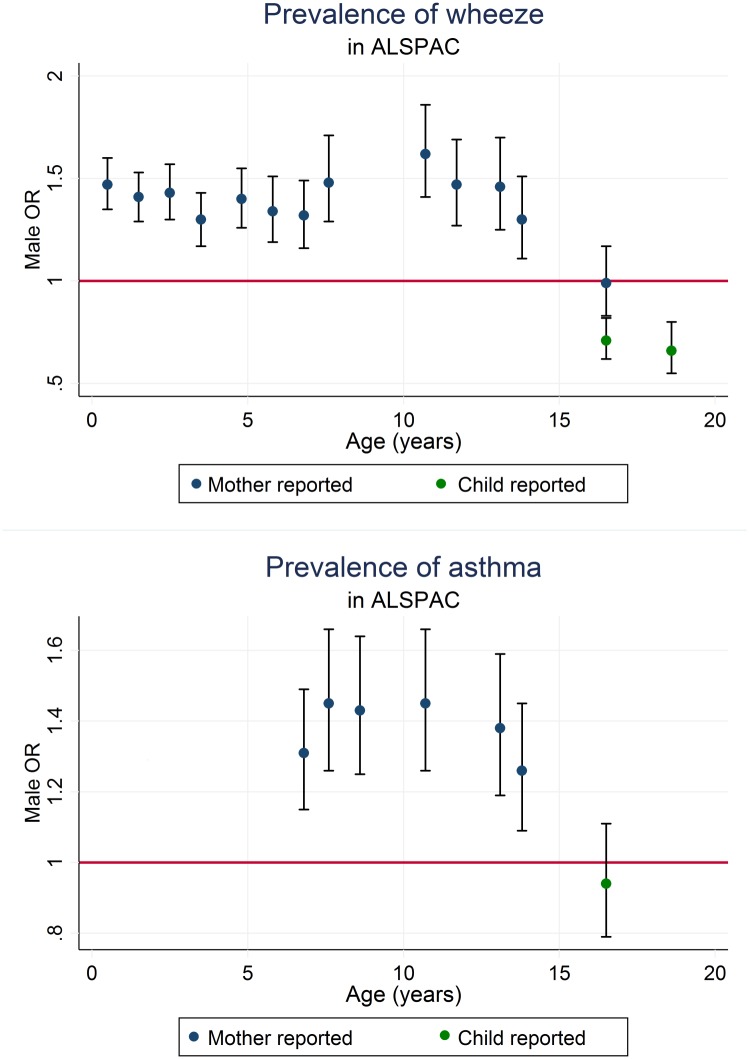
Male odds ratios for wheeze in the last 12 months and asthma in the last 12 months, in ALSPAC.

There is a higher prevalence of both asthma and wheeze in ALSPAC males up to (and including) 13.8 years where the OR is 1.26 (95% CI 1.09,1.45) for asthma in the last 12 months and 1.30 (95% CI 1.11,1.51) for (mother-reported) wheeze in the last 12 months(Fig 2 and **Table E in** S1 Appendix). A reversal of this observation can be seen at 16.5 years where the OR for males reduces to 0.99 (95% CI 0.83,1.17) when using the mother-reported wheezing in the last 12 months data and to 0.71 (95% CI 0.62,0.82) when using the child-reported wheezing data. The OR for asthma in the last 12 months using the child-reported data at 16.5 years is 0.94 (95% CI 0.79,1.11). At 18.6 years the prevalence of child-reported wheezing is lower in males with an OR of 0.66 (95% CI 0.55,0.80) for males. Results from the analysis of self-reported doctor’s diagnosis of asthma show a very similar pattern to those of the self-reported symptoms data (full results in S1 Appendix).

**Fig 3:**
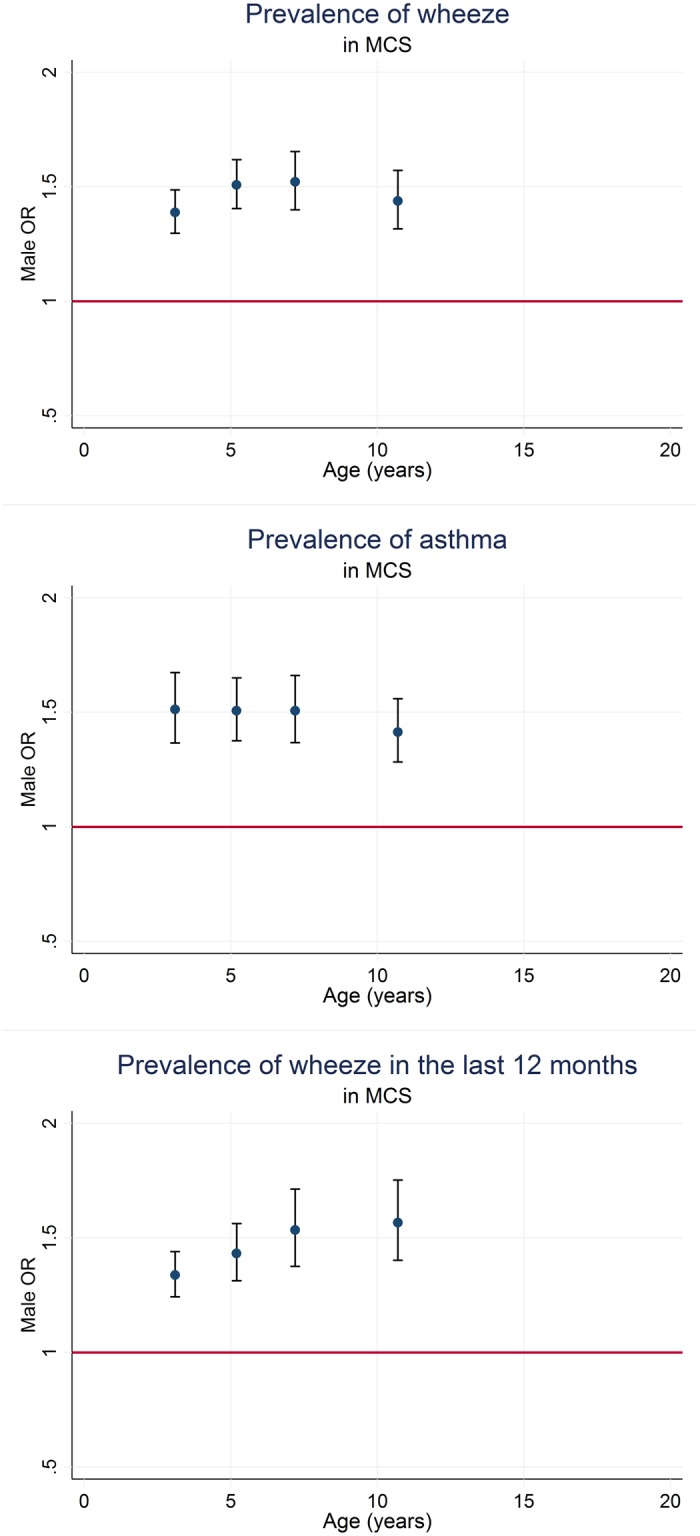
Male odds ratios for ever wheeze, ever asthma and wheeze in the last 12 months in the MCS at 4 time-points.

Cross-sectional analyses of prevalence of asthma and wheeze in the MCS show a similar pattern to that in ALSPAC (Fig 3 & **Table F in** S1 Appendix), indicating an association with sex, with a predominance in males (OR of 1.52 (95% CI 1.37,1.68) for asthma ever, 1.39 (95% CI 1.30,1.49) for wheeze ever and 1.34 (95% CI 1.25,1.45) for wheeze in the last 12 months) at age 3.1 years. The male predominance was still persistent at the last timepoint, age 10.7 years (with odds ratios of 1.42 (95% CI 1.29,1.56) for asthma ever, 1.44 (95% CI 1.32,1.58) for wheeze ever and 1.57 (95% CI 1.41,1.76) for wheeze in the last 12 months.

### Wheezing phenotypes

**Fig 4:**
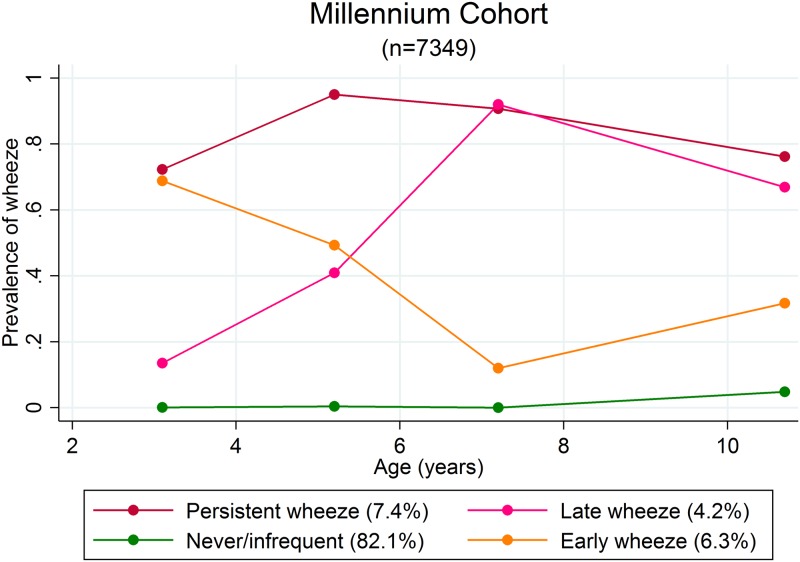
Estimated prevalence of wheezing at each time-point from 3.1 to 10.7 years for each of the four wheezing phenotypes identified by latent class analysis in 7349 participants with complete observations of wheeze in the last 12 months in the MCS.

The best fitting model based on 7349 participants with complete reports of wheezing in the last 12 months from 3.1 years to 10.7 years in the MCS resulted in 4 wheezing phenotypes (**Table H in** S1 Appendix). Trajectories of prevalence of wheeze for each of these phenotypes are presented in Fig 4. There is no Intermediate Wheezing phenotype in the MCS 4-class model, unlike the PIAMA 5-class model. The 4 classes identified represent Never/Infrequent (NI), Early Wheeze (EW), Late Wheeze (LW) and Persistent Wheeze (PW). Male:female ratios were 0.87, 1.51, 1.57 and 1.35 for each phenotype respectively.

Associations between sex and each wheezing phenotype demonstrated that males are at higher risk than females of persistent wheeze (Risk Ratio [RR] 1.73, 95% CI 1.45,2.07), late wheeze (RR 1.53, 95% CI 1.22,1.93) and early wheeze (RR 1.60, 95% CI 1.32, 1.93), when compared to never/infrequent wheeze. When early wheeze was used as the base outcome, there was no evidence that males were at higher risk of persistent wheeze (RR 1.09, 95% CI 0.84,1.40) or late wheeze (RR 0.96, 95% CI 0.72,1.28).

In the additional sex-stratified LCA model where we generated wheezing phenotypes specific to each sex in the MCS, the 4-class model remained the best fit model for both males and females based on our selection criteria (**Table J in** S1 Appendix). In the female specific models, the 3-class model indicated a marginally better fit based on BIC and entropy, however the LRT test for the 4-class model suggested that the 4-class model is indeed better than the 3-class model (p = 0.0001), with a small loss of entropy.

### Longitudinal models

**Fig 5:**
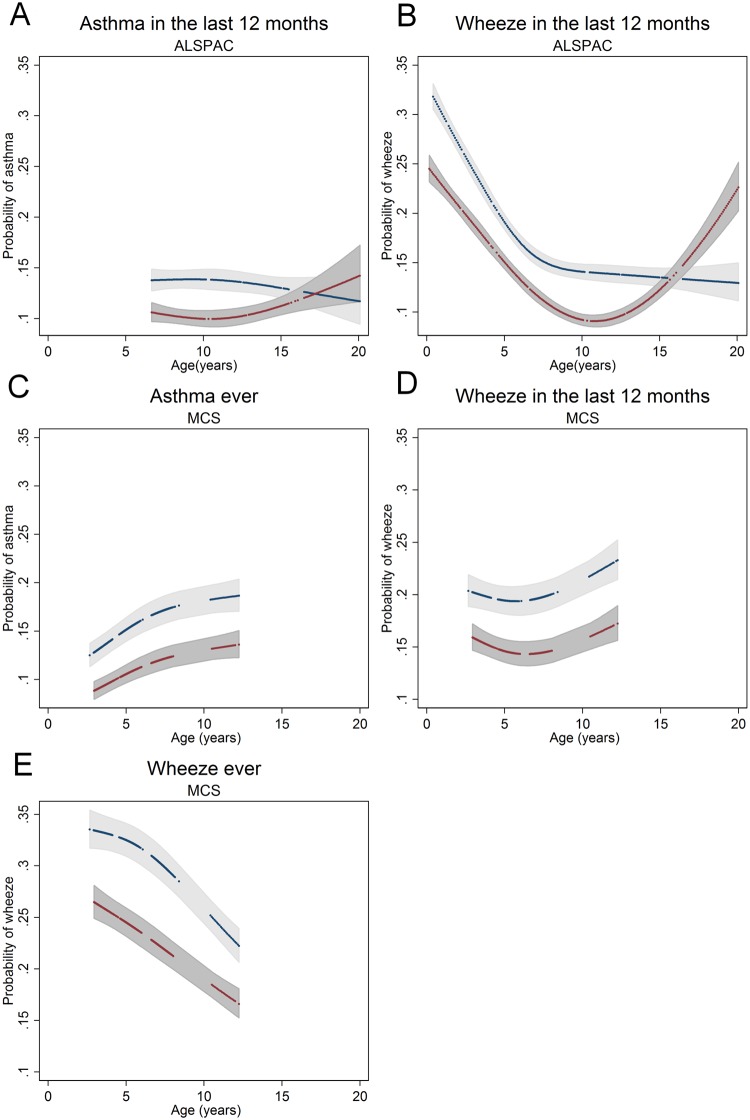
Longitudinal logistic models using cubic splines of repeated wheeze and asthma measures by sex. Males are in blue and females in red. Shaded areas represent 95% confidence intervals. Measures of (A) asthma in the last 12 months in ALSPAC (B) wheeze in the last 12 months in ALSPAC (C) asthma ever in the MCS (D) wheeze in the last 12 months in the MCS and (E) wheeze ever in the MCS.

Longitudinal models (with the association with age modelled using cubic splines) of the repeated measures of asthma and wheeze are presented in Fig 5. In ALSPAC, males start with higher prevalence of asthma (p = 0.32) than females (p = 0.25). Males have a higher probability of wheezing compared to females and both decrease with age until approx. 10 years of age. After this, males prevalence is stable at p = 0.14 while females prevalence increases to p = 0.22 at 15 years. A cross-over is evident for wheeze from male to female excess at approximately 15 years of age.

In the MCS males have a higher prevalence than females for wheeze in the last 12 months, asthma ever and wheeze ever (Fig 5) up until the last data collection point at 10 years of age. Prevalence of asthma is similar in both cohorts, however it is difficult to draw comparisons in absolute terms between the two results as the definitions used in ALSPAC for asthma are different to those in the MCS (asthma in the last 12 months vs asthma ever), and the MCS analysis includes weighting by data collection unit.

## Discussion

Results of the ALSPAC analyses appear concordant with those of the MCS analyses—cross-sectional odds ratios for asthma in the past 12 months for males at age 10.7 years are 1.45(95% CI 1.26,1.66) in ALSPAC and 1.42 (95% CI 1.29,1.56) in the MCS for ever asthma. We found no evidence of a change in the sex discordance with calendar time, as ALSPAC and MCS results were in agreement up to 10.7 years of age, at which point the MCS measurements end.

We found an increased likelihood of asthma and wheezing in males at each of the time-points, up until 16.5 years of age in the ALSPAC cohort, with little evidence of a difference at 16.5 years, where male OR was 0.99 (95% CI 0.83,1.17) for wheezing. Interestingly, in contrast to the data reported by mothers, the child-completed data at 16.5 years indicated strong evidence for a reversed prevalence in males with an OR of 0.714 (95% CI 0.62,0.82) for wheezing. These conflicting results may be due to reporting bias: parents misreporting their child’s wheeze status at 16.5 years of age, or females being more likely to self-report asthma in the child-completed questionnaire, whether correctly or incorrectly. Differences in responses between mothers and offspring can be seen **Table I in** S1 Appendix.

Overall the shift in prevalence between males and females in ALSPAC data does not occur until between 15 and 17 years of age for both asthma and wheeze. We do not observe a switch in prevalence of wheeze significantly earlier than we do for asthma, as we would expect based on evidence that wheeze is an asthma precursor [7]. However, we do see a difference at the 16.5 year time-point between asthma and wheeze, where there is strong evidence for a difference between sexes for wheeze but no evidence for a difference in asthma. It is possible that if more time-points of data existed in ALSPAC between the ages of 13.8 and 16.5 we would observe a more obvious earlier switch for wheeze. Furthermore, in the longitudinal models the same pattern is present, with a more obvious switch in wheeze than for asthma (Fig 5), with confidence intervals between males and females overlapping for asthma at 16.5 years, but not for wheeze.

As we discussed in the methods section, age at puberty cannot be a confounder in our analyses as it cannot cause a child’s sex at birth. However it is a plausible mediator of the association between sex and asthma/wheeze status. Average age at menarche in girls is estimated to be between 11.5 and 13 years and to have occurred by 14 years of age for the majority of girls [30, 31]. A secular trend towards earlier age at menarche in girls, and therefore earlier onset of puberty, has been previously reported [32–34]. Boys tend to enter puberty slightly later than girls with age at peak height velocity indicating an average age of 14 years[35]. Whereas the trend towards earlier onset of puberty has mostly been observed in girls, there is some evidence that a similar pattern is also occurring in boys [36]. Given the downward trend in the timing of puberty, we might expect to see a switch in prevalence of asthma or wheeze earlier (in ALSPAC data) than previously reported if puberty was the main mediator of the switch, and even earlier in MCS. However, our results do not suggest that a change in prevalence occurs until 16.5 years in ALSPAC, and has not been seen by age 10 years in MCS. This seems to suggest that puberty is not the main mechanism responsible. This observation is in agreement with previously published evidence that suggests that pubertal stages do not explain the shift in asthma prevalence by sex observed in adolescence [1, 37].

The question of whether puberty is causally associated with asthma status in adolescence remains. Mendelian randomization is a promising method for strengthening causal inference that uses genetic variants as instrumental variables (IVs) in order to assess causality between an exposure and outcome[38, 39]. It relies on single-nucleotide polymorphisms (SNPs) identified from genome wide association studies (GWAS) of the exposure in question. GWAS have previously found several such genetic variants that associate with pubertal onset [40]. However most associations from GWAS are relatively weak. In addition, due to the complex relationship between puberty and other anthropometric characteristics such as height, weight and body mass, as well as other environmental exposures any results are likely to not be independent of these other traits. As the Mendelian randomization framework requires independent IVs that meet certain assumption criteria [41], further work is required before such a method can be implemented to assess the effect of puberty on asthma.

Overall we did not observe the switch in prevalence of asthma/wheeze between males and females until relatively late in adolescence, indicating that puberty is unlikely to be an important factor in the switch. Although sex hormones such as estrogen and testosterone are closely correlated with timings of puberty and pubertal onset, we cannot rule out their involvement in the pathogenesis of asthma and wheeze and therefore their involvement in the shift.

If the gender switch in the prevalence of asthma is not due to puberty or changing levels of sex hormones, other mechanisms may potentially explain the change. Physical differences in the growth of lungs between sexes that change throughout development have previously been found, with boys tending to have narrower airways at birth [42]. Differences in the development of the lung structures during childhood and adolescence lead to females eventually having a smaller airway diameter in relation to lung volume in late adolescence [43]. The physiological and developmental differences of the lung could explain the gender shift seen in asthma prevalence.

### Comparisons with previous literature

As in previous studies, we found that wheeze was higher in infancy and in the first years of life, than childhood [44, 45].Overall the switch in prevalence of asthma and wheeze between sexes is not observed in our ALSPAC data until relatively late in comparison to previous studies. Cross-sectional comparisons of prevalence show greater prevalence of ever asthma in males until at least 13.8 years of age.

Longitudinal trajectories of wheezing status with other atopic symptoms have been previously identified in the MCS[46]. Using just three time-points at 3, 5 and 7 years of age, a total of four latent trajectories were identified using Latent Class Analysis. The trajectories represented low/high levels of wheezing in combination with indications of atopic symptoms. These trajectories complement our own 4-class model which are derived based on the longitudinal prevalence of wheeze and utilise 4 time-points of data. From multinomial logistic regression models of the wheezing phenotypes, both early wheeze and late wheeze males display the same increased risk, when compared to never/infrequent wheezing. Males are also at the same risk of being in the late wheeze phenotype as females when compared to the early wheeze phenotype, indicating that any switch in sex prevalence does not begin to occur until a point in time after 10.7 years. This is mirrored in previously published wheezing phenotypes derived from the ALSPAC data, where the final age time-point was at approximately 7 years of age [47].

Overall the 4-class sex-specific wheezing trajectories identified in the MCS between 3.1 and 10.7 years are in line with the 6-class wheezing trajectories stratified by sex identified previously in ALSPAC (figures A and B in S1 Appendix).The 'persistent wheeze' trajectories in MCS are comparable to the 'continuous wheeze' trajectories in ALSPAC with an initial lower probability of wheezing for the females in MCS (0.45) compared to a higher probability for the females in ALSPAC (0.80). 'Late onset wheeze' trajectories in MC are comparable to 'school-age onset persisting wheeze' trajectories in ALSPAC, where males' probability gradually increases before the females' probability also starts to increase. Finally, the 'early onset wheeze' trajectories in MC follow a similar pattern as the 'mid-childhood onset remitting wheeze' trajectories in ALSPAC, starting with high probability of wheeze and gradually declining, first males then females.

### Study strengths and limitations

The large sample size and use of novel longitudinal data from two well-characterised birth cohorts is a major strength of the study and rules out bias caused by small sample sizes.

In the ALSPAC study, loss to follow-up is associated with markers of social deprivation, increased exposure to tobacco smoke and increased reported wheeze in infancy [48]. Loss to follow-up may affect both ALSPAC and the MCS in leading to false associations between asthma and sex if sex is also related to loss to follow-up. However this is not likely to explain the changing associations with time. Whereas ALSPAC is a mostly ethnically homogenous population of individuals of Caucasian descent, MCS is a multi-ethnic population. If ethnicity affects asthma/wheeze then we would expect the incidence and prevalence rates to be different between cohorts. However, this will only affect the comparison if ethnicity modifies the association between sex and asthma/wheeze.

A further limitation is the reliance on maternal-report and self-report of symptoms from questionnaires in both cohorts, rather than clinical diagnoses of asthma/wheeze. The use of such reports may bias results away from the null if mothers or study participants are misreporting symptoms as asthma. However, the doctor-diagnosis of asthma questions, which may be considered a more reliable indication of asthma prevalence, are in broad agreement with the self-report of symptoms. Furthermore, a validation study that compared childhood asthma self-reports in ALSPAC using linked electronic patient records from GP practices showed that parental reports of a doctor's diagnosis agree well with GP-recorded diagnosis [49].

Although we have determined that there can be no confounders in our analyses, there may be effect moderators affecting the association between sex and asthma/wheeze. We have not investigated any such potential moderators.

## Conclusion

By comparing data in two large, contemporary cohorts we have shown the persistence of sex discordance in childhood asthma, with time trends suggesting that the potential narrowing of such a sex discordance is not occurring in recent cohorts, contrary to what some previously published evidence suggests. The switch in prevalence/incidence not only persists in recent collections of data but also occurs later in adolescence than would be expected if pubertal onset was the main driving factor in the switch.

## Supporting information

S1 Appendix Supporting information file. (PDF)

## Abbreviations

ALSPAC: Avon Longitudinal Study of Parents and Children
BIC: Bayesian Information Criterion
CLS: Centre for Longitudinal Studies
EW: Early Wheeze
IOW: Isle of Wight
IR: Incidence rate
ISAAC: International Study of Asthma and Allergies in Childhood
LCA: Latent Class Analysis
LLCA: Longitudinal Latent Class Analysis
LRT: Lo-Mendell-Rubin adjusted likelihood ratio test
LW: Late Wheeze
MCS: Millennium Cohort Study
NI: Never/Infrequent
OR: Odds Ratio
PIAMA: Prevention and Incidence of asthma and mite allergy birth cohort
PW: Persistent wheeze
RR: Risk Ratio
