# Supplementary material for: Sex discordance in asthma and wheeze prevalence in two longitudinal cohorts
Source: PLoS One. 2017 Apr 25;12(4):e0176293. doi: 10.1371/journal.pone.0176293 (PMC5404857; doi:10.1371/journal.pone.0176293)
Supplement: S1 Appendix — (PDF) [file pone.0176293.s001.pdf]

Table A. Data available on asthma and wheeze in ALSPAC at all time-points.

Data available		Time-point (years)														
		0.5	1.5	2.5	3.5	4.8	5.8	6.8	7.6	8.6	10.7	11.7	13.1	13.8	16.5	18.6
Mother completed questionnaires	Asthma in the past 12 months							x	x	x	x		x	x		
	Wheeze in the past 12 months	x	x	x	x	x	x	x	x	x	x	x	x	x	x	
	Doctor Diagnosis of asthma ever								x		x			x		
Child completed questionnaires	Asthma in the past 12 months														x	
	Wheeze in the past 12 months														x	x
	Doctor Diagnosis of asthma ever														x	

NUMBERS OF NEW CASES

We also estimated incidence as the number of new cases per 1000 people, based on the number of new cases of asthma or wheeze at each time point. An individual was noted as a new case at the time-point where they first respond positively to having asthma or wheeze symptoms. A sensitivity analysis using child-completed data at the 16.5 years time-point in place of the mother-completed data were also carried out. In the MCS, these estimates are not weighted for the study stratum design variable and clustering by ward is not taken into account. Due to the sampling on the MCS estimates cannot be interpreted as incidences.

For ALSPAC the number of new cases of asthma in the past 12 months show males have a higher incidence until approximately 13.8 years. There were 29.9 (95% CI 24.8,36.0) new cases per 1000 in females at age 7.6, compared to 42.8 (95% CI 36.7,49.8) new cases per 1000 in males at the same age. The trend reverses at age 13.8 years with 22.5 (95% CI 17.7, 28.5) new female cases per 1000 females compared to 14.8 (95% CI 10.9,20.0) new male. Numbers of new cases for wheeze in the past 12 months display a less clear pattern with error bars of females and males overlapping at multiple time-points. Numbers of new cases for wheeze in the last 12 months start high (>170 cases per 1000 individuals) in the first 2 years of life and then steadily decline. By age 18.6 years females have a markedly higher number of new cases than males at 129 (95% CI 110.6,150.1) per 1000 females compared to 58.1 (95% CI 40.4,83.0) per 1000 males. A sensitivity analysis using child-completed data at the 16.5 years time-point in place of the mother-completed data yielded very similar results.

The number of new cases in the MCS calculated for asthma ever, wheeze ever and wheeze in the last 12 months are higher for males in both measures until 7.2 years, with the number of new cases at 5.2 years being 93.7 (95% CI 76.4,114.4) per 1000 males and 66.2 (95% CI 55.3,79.2) per 1000 females for asthma ever. However the difference between males and females becomes progressively smaller. By 10.7 years the difference appears smaller with 62.1 (95% CI 54.2,71.0) new cases per 1000 males and 49.7 (95% CI 49.7,55.1) per 1000 females for “asthma ever”. Similarly for both “wheeze in the last 12 months” and “wheeze ever”, where at 10.7 years, there were 81.2 (95% CI 71.1,92.7) new cases per 1000 males and 65 (95% CI 57.7,73.3) per 1000 females for wheeze ever.

Table B. Numbers of new cases for asthma in the last 12 months in ALSPAC at each time-point per 1000 individuals.

	Asthma last 12 months	
Age(years)	Males [95% CI]	Females [95% CI]
7.6	42.77 [36.69,49.79]	29.9 [24.81,35.98]
8.6	38.66 [32.81,45.51]	24.87 [20.24,30.51]
10.7	41.48 [35.14,48.9]	30.14 [24.88,36.47]
13.1	31.37 [25.52,38.5]	24.97 [19.96,31.21]
13.8	14.75 [10.88,19.97]	22.47 [17.69,28.5]
16.5*	26.98 [20.06,36.19]	39.94 [32.87,48.46]

* offspring self-report

Table C. Numbers of new cases for wheeze in the last 12 months in ALSPAC at each time-point per 1000 individuals.

	Wheeze last 12 months	
Age (years)	Males [95% CI]	Females [95% CI]
1.5	208.06 [195.93,220.72]	173.28 [162.14,185.01]
2.5	128.12 [116.71,140.46]	97.72 [87.97,108.43]
3.5	73.41 [64.17,83.86]	62.84 [54.64,72.18]
4.8	74.89 [64.96,86.19]	50.08 [42.4,59.06]
5.8	42.95 [34.98,52.65]	41.46 [34.05,50.39]
6.8	25.93 [19.7,34.05]	24.53 [18.83,31.9]
7.6	21.14 [15.54,28.69]	19.72 [14.61,26.59]
8.6	52.58 [43.44,63.52]	30 [23.64,38.02]
10.7	38.15 [30.02,48.37]	25.45 [19.44,33.25]
11.7	33.71 [25.9,43.77]	30.7 [23.9,39.34]
13.1	19.92 [13.87,28.53]	20.9 [15.24,28.59]
13.8	15.98 [10.64,23.94]	17.97 [12.73,25.31]
16.5	14.42 [8.85,23.42]	40.66 [31.68,52.03]
18.6*	58.1 [40.36,82.96]	129.04 [110.59,150.05]

* Offspring self-report

Table D. Numbers of new cases for asthma and wheeze in the MCS at each time-point per 1000 individuals.

	Asthma ever		Wheeze ever		Wheeze in the last 12 months	
Age (years)	Males [95% CI]	Females [95% CI]	Males [95% CI]	Females [95% CI]	Males [95% CI]	Females [95% CI]
5.2	93.7 [76.4,114.4]	66.2 [55.3,79.2]	199.1 [178.1,221.9]	147.9 [128.7,169.3]	141.4 [167.8,118.5]	106.1 [124.6,90.1]
7.2	52.4 [46.1,59.5]	38.7 [33.4,44.8]	88.4 [75.4,103.4]	69.4 [59.6,80.6]	70.4 [84.2,58.7]	51.5 [63.4,41.7]
10.7	62.1 [54.2,71]	49.7 [44.9,55.1]	81.2 [71.1,92.7]	65 [57.7,73.3]	87.3 [102.4,74.3]	70 [85.1,57.4]

Table E. Prevalence of asthma and wheeze in the last 12 months by sex modelled cross-sectionally across the available childhood time-points in ALSPAC.

	Male OR [95% CI] for asthma in last 12 months*	Male OR [95% CI] for wheeze in last 12 months*
Mother completed time-points (years)		
0.5	NA	1.46 [1.35, 1.60]
1.5	NA	1.41 [1.29, 1.53]
2.5	NA	1.43 [1.30, 1.57]
3.5	NA	1.29 [1.16, 1.43]
4.8	NA	1.39 [1.25, 1.55]
5.8	NA	1.34 [1.19, 1.51]
6.8	1.31 [1.15, 1.49]	1.32 [1.16, 1.49]
7.6	1.45 [1.26, 1.66]	1.48 [1.29, 1.71]
8.6	1.43 [1.25, 1.64]	NA
10.7	1.45 [1.26, 1.66]	1.62 [1.41, 1.86]
11.7	NA	1.47 [1.27, 1.69]
13.1	1.38 [1.19, 1.59]	1.46 [1.25, 1.70]
13.8	1.26 [1.09, 1.45]	1.30 [1.11, 1.51]
16.5	NA	0.99 [0.83, 1.17]
Child completed time-points (years)		
16.5	0.94 [0.79, 1.11]	0.71 [0.62, 0.83]
18.6	NA	0.66 [0.55, 0.80]

* In last 6 months for 1st time-point

Table F. Prevalence of asthma ever, wheeze ever and wheeze in the last 12 months by sex modelled cross-sectionally across the available childhood time-points in the Millennium Cohort Study.

	Male OR [95% CI] Asthma ever	Male OR [95% CI] Wheeze ever	Male OR [95% CI] Wheeze in last 12 months
Time-points (years)			
3.1	1.52 [1.37,1.68]	1.39 [1.3,1.49]	1.34 [1.25,1.45]
5.2	1.51 [1.38,1.66]	1.51 [1.41,1.62]	1.44 [1.32,1.57]
7.2	1.51 [1.37,1.67]	1.53 [1.4,1.66]	1.54 [1.38,1.72]
10.7	1.42 [1.29,1.56]	1.44 [1.32,1.58]	1.57 [1.41,1.76]

Table G. Prevalence of asthma as defined by doctor diagnosis of asthma ever by sex modelled cross-sectionally across the 4 available childhood time-points in ALSPAC.

Time-point (years)	Male OR [95% CI] for doctor diagnosis asthma
7.6	1.36[1.22,1.51]
10.7	1.42[1.27,1.58]
13.8	1.25[1.12,1.40]
Child-completed time-points (years)	
16.5	1.13 [0.99,1.28]

Table H. Statistics of the different wheezing phenotypes models attempted in the MCS, used to select the best fitting model.

	3-class	4-class	5-class	6-class
Number of parameters	14	19	24	29
BIC	15601	15586.074	15630.585	15675.097
Entropy	0.953	0.894	0.893	0.966
Lo-Mendell-Rubin Likelihood Ratio Test (Adjusted p-value)	<0.0001	<0.0001	1.00	0.50

Table I. Differences between the child-completed self-reports of wheeze in the last 12 months at 16.5 years compared to the mother-completed questionnaires at the same age in ALSPAC, with percentage of total responders in each sex.

	Child-completed			
Mother - completed	Male		Female	
	No	Yes	No	Yes
No	1,427(80.4%)	175(9.9%)	1,737(76%)	297(13%)
Yes	44(2.5%)	128(7.2%)	56(2.5%)	195(8.5%)

Table J. Statistics of the different wheezing phenotypes models attempted in the MCS when stratified by sex.

Females				
	3-class	4-class	5-class	6-class
Number of parameters	14	19	24	29
BIC	6873	6896.217	6937.396	6978.576
Entropy	0.946	0.894	0.946	0.969
Lo-Mendell-Rubin Likelihood Ratio Test (Adjusted p-value)	0.0015	0.0001	0.50	0.50
Males				
	3-class	4-class	5-class	6-class
Number of parameters	14	19	24	29
BIC	8717	8715.878	8756.787	8797.696
Entropy	0.938	0.909	0.916	0.586
Lo-Mendell-Rubin Likelihood Ratio Test (Adjusted p-value)	<0.0001	<0.0001	0.46	0.53

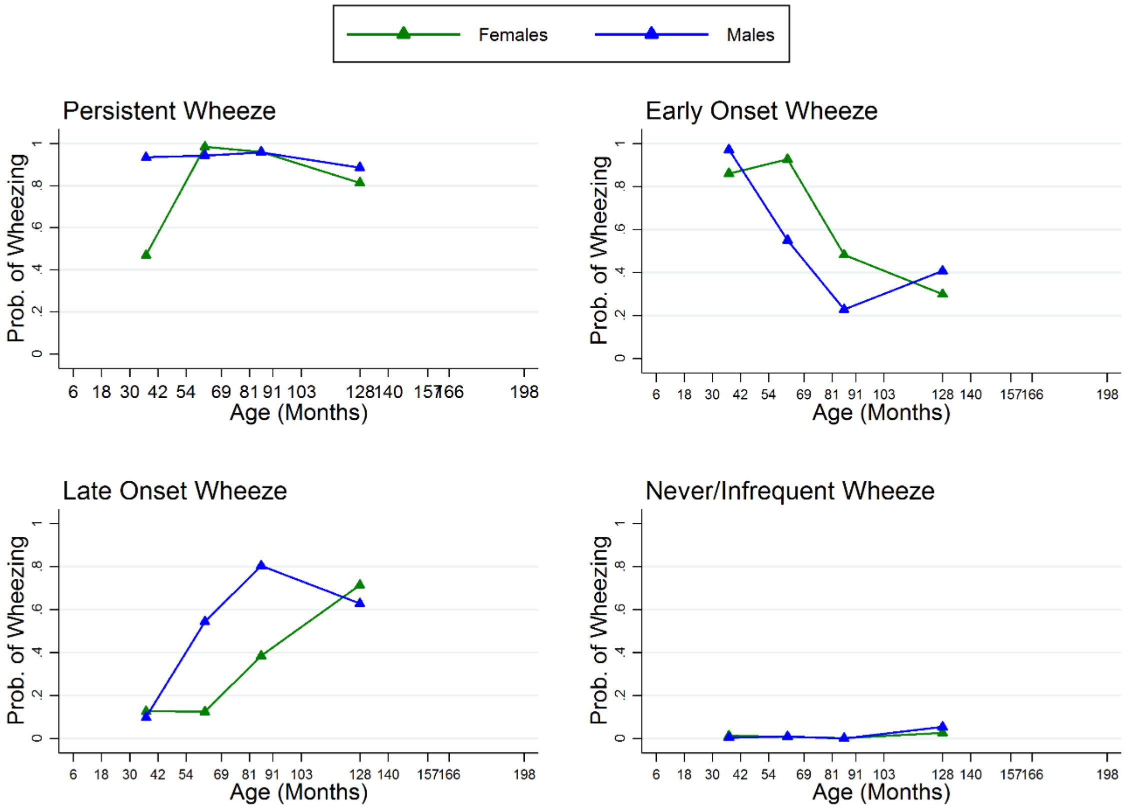

Figure A. Estimated prevalence of wheezing at each time-point (from 3.1 to 10.7 years) for each of the four wheezing phenotypes, where green lines represent females and blue lines males. Figure is adapted to match Figure S4 reproduced from *Granell et al. JACI 2016* with permission, for comparison.

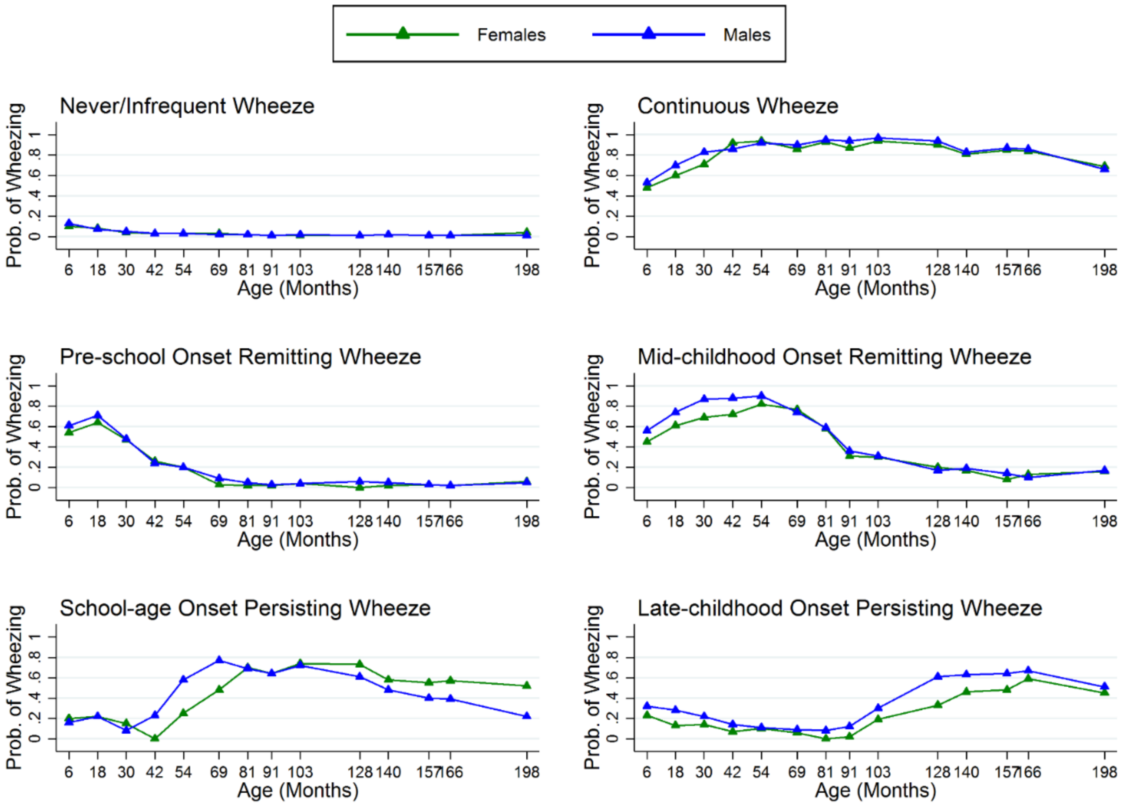

Figure B. Reproduced with permission from *Granell et al. JACI 2016 Figure 2*. Estimated prevalence of wheezing at each time point from birth to 16½ years for each of the 6 wheezing phenotypes identified by using latent class analysis in 12,303 participants with at least 2 observations of wheeze by sex.

Table K. Longitudinal logistic model of repeated wheeze and asthma measures using restricted cubic splines in ALSPAC and the MCS, adjusted interactions of sex with age in each spline.

	**Prevalence [95% CI]**	**P-value**
**ALSPAC†**		
**Wheeze 12 months†**		
0.5-3.5 years	0.894 [0.86,0.93]	0.001
3.5-6.8 years	0.821 [0.567,1.189]	0.295
6.8-10.7 years	1.735 [0.656,4.59]	0.267
10.7-16.7years	1.084 [0.424,2.772]	0.867
Sex*age (0.5-3.5 years)	0.973 [0.924,1.024]	0.285
Sex*age (3.5-6.8 years)	1.068 [0.652,1.749]	0.797
Sex*age (6.8-10.7 years)	1.352 [0.369,4.958]	0.650
Sex*age (10.7-16.7years)	0.275 [0.078,0.971]	0.045
Sex**	1.495 [1.345,1.662]	0.001
**Asthma 12 months†**		
6.8-10.8 years	0.974 [0.94,1.009]	0.136
10.8-16.6 years	1.069 [1.011,1.131]	0.020
Sex*age (6.8-10.8 years)	1.032 [0.984,1.083]	0.201
Sex*age (10.8-16.7 years)	0.915 [0.847,0.989]	0.024
Sex**	1.094 [0.723,1.657]	0.671
**MCS††**		
**Wheeze ever††**		
3.1-5.5 years	0.953 [0.926,0.981]	0.001
5.5-11.3 years	0.974 [0.923,1.028]	0.329
Sex *age (3.1-5.5 years)	1.034 [0.994,1.075]	0.098
Sex*age (5.5-11.3 years)	0.947 [0.881,1.019]	0.142
Sex**	1.261 [1.058,1.504]	0.010
**Asthma ever††**		
3.1-5.5 years	1.107 [1.065,1.151]	0.001
5.5-11.3 years	0.916 [0.855,0.981]	0.012
Sex *age (3.1-5.5 years)	0.999 [0.95,1.051]	0.963
Sex*age (5.5-11.3 years)	0.995 [0.909,1.089]	0.899
Sex**	1.524 [1.2,1.935]	0.001
**Wheeze 12 months††**		
3.1-5.5 years	0.945 [0.912,0.979]	0.002
5.5-11.3 years	1.132 [1.06,1.21]	0.001
Sex *age (3.1-5.5 years)	1.028 [0.981,1.078]	0.26
Sex*age (5.5-11.3 years)	0.968 [0.886,1.058]	0.462
Sex**	1.228 [0.999,1.51]	0.052

* Change in prevalence of asthma/wheeze per 1 year increase in age.

** Change in prevalence of asthma/wheeze for males compared to females.

†Measures used in the ALSPAC longitudinal models are wheeze in the last 12 months from 15 time-points and asthma in the last 12 months from 7 time-points.

†† Measures from the Millennium Cohort Study used in the longitudinal models are ever wheeze, ever asthma and wheeze in the last 12 months from 4 time-points.
